# RT-qPCR Assays for Rapid Detection of the N501Y, 69-70del, K417N, and E484K SARS-CoV-2 Mutations: A Screening Strategy to Identify Variants With Clinical Impact

**DOI:** 10.3389/fcimb.2021.672562

**Published:** 2021-05-20

**Authors:** Natali Vega-Magaña, Rocío Sánchez-Sánchez, Jorge Hernández-Bello, Alberto Antony Venancio-Landeros, Marcela Peña-Rodríguez, Rosa Alejandra Vega-Zepeda, Byron Galindo-Ornelas, Mauricio Díaz-Sánchez, Mariel García-Chagollán, Gabriela Macedo-Ojeda, Octavio Patricio García-González, José Francisco Muñoz-Valle

**Affiliations:** ^1^ Institute for Research in Biomedical Sciences (IICB), University Center for Health Sciences, University of Guadalajara, Guadalajara, Mexico; ^2^ Laboratory for the Diagnosis of Emerging and Reemerging Diseases (LaDEER), University Center for Health Sciences, University of Guadalajara, Guadalajara, Mexico; ^3^ Research and Development Department, Genes2life (Grupo T), Irapuato, Mexico; ^4^ Bioinformatics Department, Genes2life (Grupo T), Irapuato, Mexico

**Keywords:** SARS-CoV-2, E484K, P.2 variant detection, SARS-CoV-2 mutations, SARS-CoV-2 mutation screening, molecular screening, epidemiological surveillance

## Abstract

**Background:**

Several variants of the SARS-CoV-2 have been documented globally during the current COVID-19 pandemic. The N501Y, 69-70del, K417N, and E484K SARS-CoV-2 mutations have been documented among the most relevant due to their potential pathogenic biological effects. This study aimed to design, validate, and propose a fast real-time RT-qPCR assay to detect SARS-CoV-2 mutations with possible clinical and epidemiological relevance in the Mexican population.

**Methods:**

Targeting spike (*S*) gene mutations of SARS-CoV-2 (N501Y, 69-70del, K417N, and E484K), specific primers, and probes for three specific quantitative reverse transcription PCR (RT-qPCR) assays were designed, and validated using Sanger sequencing. These assays were applied in clinical samples of 1060 COVID-19 patients from Jalisco Mexico.

**Results:**

In silico analyzes showed high specificity of the three assays. Amplicons of samples were confirmed through sequencing. The screening of samples of COVID-19 patients allowed the identification of the E484K mutation in nine individuals and the identification of P.2 Brazilian variant in Mexico.

**Conclusion:**

This work provides low-cost RT-qPCR assays for rapid screening and molecular surveillance of mutations with potential clinical impact. This strategy allowed the detection of E484K mutation and P.2 variant for the first time in samples from the Mexican population.

## Introduction

COVID-19 is an infectious disease, being first identified in China towards the end of December 2019. Nowadays, COVID-19 has become a growing pandemic. The etiological agent of this disease was initially named Novel Coronavirus 2019 (2019-nCoV) by the Chinese Center for Disease Control and Prevention (CDC) (Khan et al., 2020; Zhu et al., 2020) and was subsequently renamed Severe Acute Respiratory Syndrome-Coronavirus-2 (SARS-CoV-2) due to its homology with SARS-CoV (Mehta et al., 2020).

SARS-CoV-2 is a type of Betacoronavirus, considered to have the second largest genome of all RNA viruses with a 5’ cap and 3’ poly-A tail. Phylogenetic analyses of coronaviruses reveal that SARS-CoV-2 is 96% genetically related to the Bat-SARS Like-Corona virus (Bat-SL-Cov) (Zhou et al., 2020).

The ORF1ab and ORF1a at the 5’ SARS-CoV-2 terminal region of the genome encode the 1ab and 1a polypeptides, which are proteolytically cleaved into 16 different nonstructural proteins (NSPs). The 3’ terminal of the genome represents four structural (spike, envelope, matrix, and nucleocapsid) and nine accessory proteins (3a, 3b, 6, 7a, 7b, 8b, 9a, 9b, and orf10) (Al-Qaaneh et al., 2021, 2).

The “spike protein” is the characteristic glycoprotein family present in the surface of the coronavirus, giving it the appearance of a crown when observed through electron microscopy (EM), hence the name “corona-virus”, coming from the Latin *crown*. The spike protein of SARS-CoV-2 binds to the angiotensin-converting enzyme 2 (ACE2) receptor to expose the cleavage sites to cellular proteases, to initiate fusion endocytosis with the host cell. Therefore, this protein has become an important target for vaccine development, blocking therapy with antibodies and diagnostic antigen-based tests (Pillay, 2020).

After the first SARS-CoV-2 genomic sequence was delivered (Wu et al., 2020), several research groups reported an accelerated genetic evolution of SARS-CoV-2 through phylogenetic analysis (Rambaut et al., 2020; Yi, 2020; Fiorentini et al., 2021).

New variants of SARS-CoV-2 are spreading worldwide rapidly, becoming a global threat. Three variants are the most notable due to their pathogenic potential: The United Kingdom variant (called B.1.1.7), with the potential to spread more easily and quickly than other reported variants; and also the South Africa (called B.1.351) and Brazil (called P.1 and P.2) variants, which are being studied due to their potential to affect the efficiency of the SARS-CoV-2 neutralizing antibodies (CDC, 2020; Tegally et al., 2020; Voloch et al., 2020; Arif, 2021; Francisco et al., 2021; Galloway et al., 2021; Wu et al., 2021).

Four mutations (N501Y, 69-70del, K417N, and E484K) in the spike protein could explain the potential biological effects that have been described for these variants. Mutation N501Y has been found on the receptor-binding domain (RBD) and has been associated with an increase of binding affinity to the ACE2 receptor (Starr et al., 2020; Leung et al., 2021). Additionally, the 69-70del, K417N, and E484K mutations have been described as possible “escape mutations,” in the context of their association with the humoral immune response evasion (Andreano et al., 2020; Fratev, 2020; Bal et al., 2021; Sabino et al., 2021; Xie et al., 2021).

Full genome sequencing is the gold standard for identifying SARS-CoV-2 variants; however, this methodology is not available to most developing countries. Understanding the introduction, spread, and establishment of potential pathogenic SARS-CoV-2 mutations is crucial to enable effective control strategies. Thus, this study aimed to design, standardize, and propose a low-cost RT-qPCR assay to detect SARS-CoV-2 mutations with pathogenic effects in the Mexican population.

## Materials and Methods

### Bioinformatic Processing

The SARS-CoV-2 sequences were downloaded from the GISAID database (Elbe and Buckland-Merrett, 2017). For the design of the assays, the genomes considered were reported from samples collected between December 16, 2020, and January 15, 2021. From these, all loci corresponding to the spike glycoprotein were extracted, comprised between nucleotides 21,563 to 25,384 in the reference genome of SARS-CoV-2 (NCBI Reference Sequence: NC_045512.2). We considered the slight spike locus variation due to insertions or deletions in the process of extracting this region from the other genomes.

Once all the sequences corresponding to the spike protein had been obtained (a total of 31,357), the grouping of sequences was accomplished using CD-HIT (Li et al., 2001; Fu et al., 2012), applying a cut-off of 100% identity. A total of 5,422 clusters were obtained, each with a unique representative sequence. These sequences were aligned using MAFFT (Katoh et al., 2002).

The alignment produced by MAFFT was revised thoroughly with UGENE (Okonechnikov et al., 2012) and MEGA7 (Kumar et al., 2016) software. Once we ensured that the alignment did not contain any errors, it was taken to elaborate the consensus sequences of the regions of interest, in order to design primers and probes.

### Assays, Primers, and Probes

Due to the heterogeneity of the mutations to be detected, three tests were developed, including the detection of the mutations of interest regarding the gene encoding for the SARS-CoV-2 spike protein. The 69/70 deletion test is aimed at the discrimination of sequences that contain the deletion of amino acids 69 and 70, while the K417N test detects a single base substitution that causes the exchange of a Lysine (K) for an Asparagine (N), and the E484K/N501Y assay detects the single base mutations that exchange Glutamic Acid (E) for Lysine (K) at position 484, while also detects the change of an Asparagine (N) for a Tyrosine (Y) at position 501.

Primers and probes were designed from the consensus sequences obtained from the sequence alignment for each region of interest (**Table 1**). OligoCalc software (Kibbe, 2007) was used for the calculation of melting temperature (Tm), while for the prediction of secondary structures, the tool chosen was OligoAnalyzer software (https://www.idtdna.com/calc/analyzer). These oligonucleotides and probes were synthesized by the company T4 Oligo.

**Table 1:** Sequences of probes and primers designed for the mutation detection assays.

Assay	Oligonucleotide names	oligonucleotide sequences	Modifications	Tm °C
69/70 deletion	Del 69/70 FW	GACTTGTTCTTACCTTTCTTTTCC		60.3
	Del 69/70 RV	CATCATTAAATGGTAGGACAGGG		60.9
	Probe 69/70	TCCATGCTATACA(T)GTCTCTGGGACCAAT	3´C3 T-BHQ1 5´FAM	69.1
	Probe Del 6970	GTTCCATGCTATC(T)CTGGGACCAATGGT	3´C3 T-BHQ2 5´Cal Fluor Red 610	70.1
K417N mutation	K417N RV	AATTACCACCAACCTTAGAATCAAG		60.9
	417K FW	CTCCAGGGCAAACTGGAAA+G	G LNA	65
	417N FW	GCTCCAGGGCAAACTGGAAA+T	T LNA	66
	Probe K417N	CCAGATGATTTTACAGGCTGCGTTATAG	3´BHQ3 5´Quasar 670	67
E484K/N501Y mutations (duplex-assay)	484/501 FW	ATCTATCAGGCCGGTAGCAC		60.5
	484/501 RV	GTACTACTACTCTGTATGGTTGG		60.9
	Probe 484E	CTTGTAATGGTGTTGAAGGTTTTAATTG	3´BHQ1 5´FAM	62.7
	Probe 484K	CTTGTAATGGTGTTAAAGGTTTTAATTG	3´BHQ2 5´Cal Fluor Red 610	61.5
	Probe 501N	TCCAACCCACT+AATGGTGTTGG	3´BHQ1 A LNA 5´HEX	66
	Probe 501Y	TCCAACCCACT+TATGGTGTTGG	3´BHQ3 T LNA 5´Quasar 670	66

The letter “T” in parentheses denotes the position of the internal quencher. The symbol “+” indicates that the next base is a modified base (LNA).

### Controls and Standardization

For the standardization of the assays, the viral RNA was taken from clinical samples (nasopharyngeal swabs) of previously diagnosed COVID-19 patients, using the PureLink Viral RNA kit (Invitrogen), following the manufacturer’s recommendations. Clinical samples were inactivated and manipulated using personal protective equipment according to the CDC recommendations, which are available at https://www.cdc.gov/coronavirus/2019-ncov/lab/guidelines-clinical-specimens.html

The presence of viral RNA in the extracts was verified by performing RT-qPCR using the DeCoV19 Kit Triplex kit (Genes2Life SAPI de CV). On the other hand, due to the lack of viral RNA sequences with the mutations to be detected, we used double-stranded DNA controls (provided by ADN SINTETICO SAPI de CV) containing the regions of interest (**Table 2**).

**Table 2:** Synthetic DNA controls for tests.

Control	Control sequences	Mutations present in the sequence
C+ without-Del69-70	GACTTGTTCTTACCTTTCTTTTCCAATGTTACTTGGTTCCATGCTATA**CATGTC**TCTGGGACCAATGGTACTAAGAGGTTTGATAACCCTGTCCTACCATTTAATGATGGTGTTTATTTTGCTTCCACTGAGAAGTCTAACATAATAAGAGGCTGGATTTTTGGT	None
C+ Del69-70	GACTTGTTCTTACCTTTCTTTTCCAATGTTACTTGGTTCCATGCTATATCTGGGACCAATGGTACTAAGAGGTTTGATAACCCTGTCCTACCATTTAATGATGGTGTTTATTTTGCTTCCACTGAGAAGTCTAACATAATAAGAGGCTGGATTTTTGGT	69/70 deletion
C+ without Mut484-501-417	GCTCCAGGGCAAACTGGAAA**G**ATTGCTGATTATAATTATAAATTACCAGATGATTTTACAGGCTGCGTTATAGCTTGGAATTCTAACAATCTTGATTCTAAGGTTGGTGGTAATTATCTATCAGGCCGGTAGCACACCTTGTAATGGTGTT**G**AAGGTTTTAATTGTTACTTTCCTTTACAATCATATGGTTTCCAACCCACTAATGGTGTTGGTTACCAACCATACAGAGTAGTAGTAC	None
C+ Mut484-501-417	GCTCCAGGGCAAACTGGAAA**T**ATTGCTGATTATAATTATAAATTACCAGATGATTTTACAGGCTGCGTTATAGCTTGGAATTCTAACAATCTTGATTCTAAGGTTGGTGGTAATTATCTATCAGGCCGGTAGCACACCTTGTAATGGTGTT**A**AAGGTTTTAATTGTTACTTTCCTTTACAATCATATGGTTTCCAACCCACT**T**ATGGTGTTGGTTACCAACCATACAGAGTAGTAGTAC	K417N E484K N501Y

C+ = positive control; changes in sequences of controls are underlined in bold.

### RT-qPCR Assay Design: Alignment and Discrimination Site

#### 69/70 Deletion Assay

**Figure 1:**
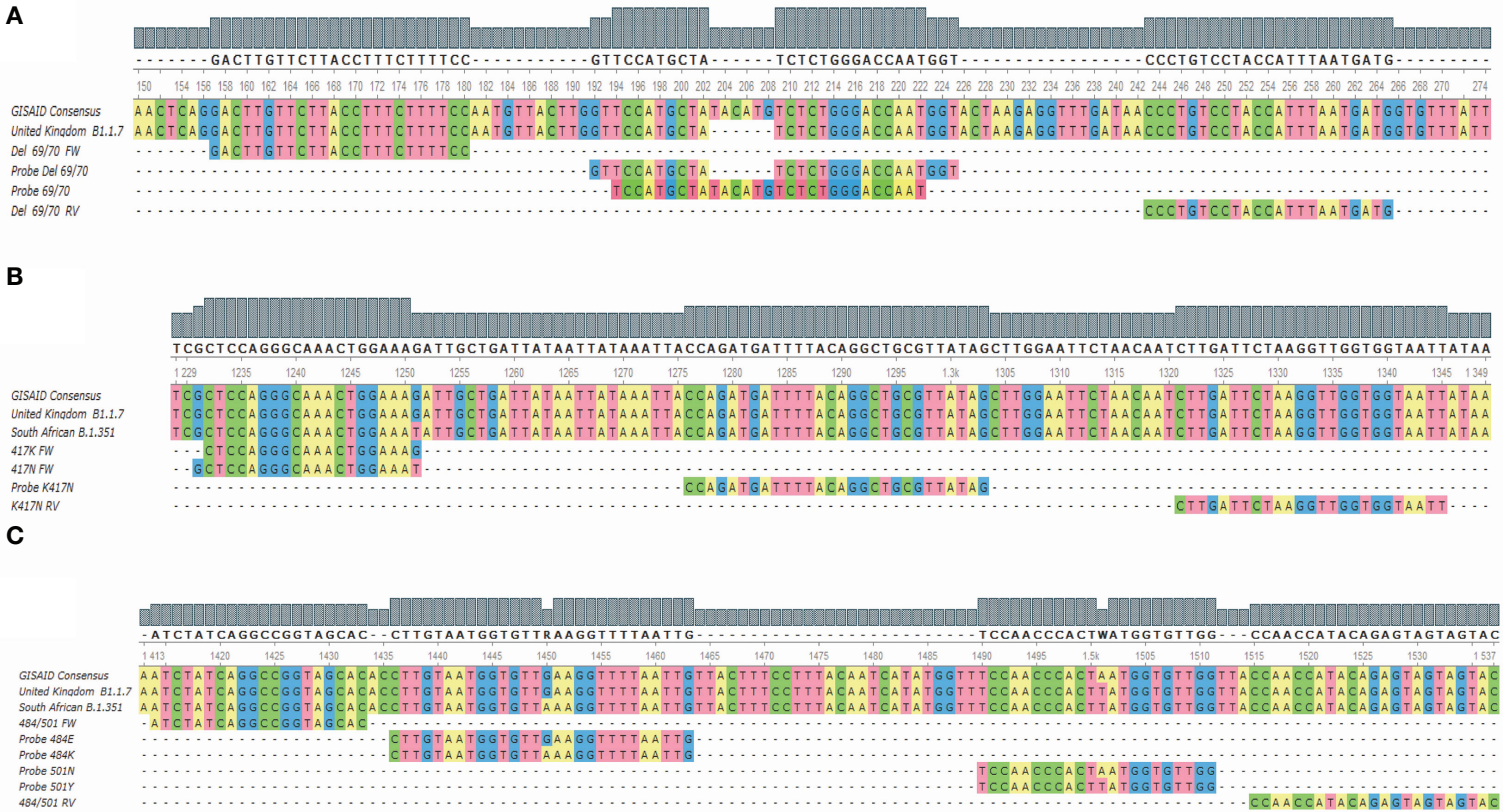
Alignment of the hybridization regions of the oligonucleotides and the probes with the target sequences for the three assays. **(A)** Shows the alignment for the 69/70 deletion assay; **(B)** shows the alignment for the K417N mutation assay; **(C)** shows the alignment for the E484K/N501Y assay. FW, Forward; RV, reverse.

This assay is directed to the region where the 69/70 deletion from the coding sequence for the SARS-CoV-2 spike is found and has a set of primers that allow the amplification of both mutated and non-mutated sequences. The probe “Probe del69/70”, labeled with CFR 610 perform the detection of the viral RNAs that have the deletion, and the probe “Probe 69/70” (**Table 1**), labeled with FAM to detect the sequences that do not present the deletion (**Figure 1A**), allowing the sequence discrimination in a single reaction.

#### K417N Mutation Assay

The region corresponding to the K417N mutation presents a high complexity at designing standard probes that allow the sequence discrimination. Therefore, it was decided to carry out the discrimination in two independent reactions. We made use of both a probe and a reverse primer that both do not discriminate between mutant and non-mutant sequences, as well as a forward primer “417K FW” for the first reaction, where the sequences without the mutation are detected; and the primer “417N FW” for the second reaction, where the sequences that present the mutation are detected (**Figure 1B**). Both primers (“417K FW” and “417N FW”) were modified at the 3’ end with a locked nucleic acid (LNA) base to increase the variations of the melting temperature (ΔTm) of the hybridization between the complementary base, while mismatching to favor the hybridization of the appropriate primer.

#### E484K/N501Y Assay

The E484K and N501Y mutations are single nucleotide variants close to each other. This allowed the design of an assay that uses a set of primers that amplify both the sequences, containing the mutations or the wild-type base. Therefore, this assay uses four fluorescent probes that specifically hybridize with the target sequences to discriminate each base/mutation (**Figure 1C**). Thus, the detection and discrimination were carried out in a quadruplex assay, being the channels for the FAM and HEX fluorophores where the sequences without E484K and N501Y mutations will be detected through the probes “Probe 484E” and “Probe 501N”, respectively. E484K and N501Y mutations were detected in the Cal Fluor Red 610 (Probe 484K) and Quasar 670 (Probe 501Y) channels, respectively.

As the probes differ only on a single nucleotide, there will be hybridization competition during the alignment and extension phase of thermocycling, favoring the specific binding of probes that do not have a mismatch with their target (Jacobsen et al., 2002). For the N501Y mutation, modified bases (LNA) were used to allow better discrimination due to the low ΔTm of hybridization between the complementary base and the mismatch.

### Assay Standardization

The standardization of the tests was performed using the synthetic controls C+ Del69-70, C+ Mut484-501-417, C+ without-Mut484-501-417, and C+ without-Del69-70 (**Table 2**), as well as RNA extracted from samples that tested positive to SARS-CoV-2 (previously detected by PCR test) and corroborated with the DeCoV19 Kit Triplex molecular diagnostic kit (Genes2Life SAPI de CV).

Detection assays were carried out in parallel with the StarQ One-Step RT-qPCR kit enzyme (Genes2Life SAPI de CV) and the SuperScript III Platinum One-Step qRT-PCR System enzyme (Invitrogen) using reactions with a final volume of 25µL. Although the results of assays 69/70 and E484K/N501Y and K417N were comparable with both enzymes, we had difficulties using the enzyme SuperScript III Platinum One-Step qRT-PCR System because of dimer primer formation; therefore, its use is not recommended for these assays.

The optimization of the cycling temperatures for each assay was carried out in the CFX96 Deep Well Real-Time System (BIO-RAD) by using different temperature gradients for the reverse transcription steps, with a temperature range from 48 to 55°, using RNA from clinical samples. For the alignment/extension step conditions optimization, RNA and the synthetic controls corresponding to each assay were used (See **Table 2**), considering the Tm of the primers and probes designed for each assay as a starting point. The optimization of this step included temperatures from 55°C and up to 68°C. In the same way, primers and probes concentrations were optimized applying concentration variations; all of the above is summarized in **Table 3**.

**Table 3:** Reaction and amplification conditions of the assays.

Assay	Primer/Probe	Concentration/reaction (µM)	Cycling conditions	
69/70 Del	Del 69/70 FW	0.8	1X	52°C, 30 min
	Del 69/70 RV	0.8	1X	95°, 3min
	Probe 69/70	0.2	45X	95 °C, 15 sec
	Probe Del 69/70	0.22		60°C, 30 sec*
K417N	K417N RV	0.8	1X	50°C,15 min
	417K FW	0.8	1X	95°C, 3min
	417N FW	0.8	45X	95°C, 15 sec
	Probe K417N	0.2		67°C, 30 sec*
E484K/N501Y	484/501 FW	0.8	1X	50°C 30 min
	484/501 RV	0.8	1X	95°C 5min
	Probe 484E	0.2	45X	95°C 15 sec 62°C 30 sec*
	Probe 484K	0.28		
	Probe 501N	0.2		
	Probe 501Y	0.28		

1X, 1 cycle; FW, forward; RV, reverse; * Denotes the step for fluorescence reading.

An important difference that is worth to be noted is that standard diagnostic assays select the optimal temperature like the one in which the assay has the highest efficiency. In contrast, in our assays, the selected temperature was the one that allowed us to differentiate the amplification of wild-type and mutant samples more clearly. Therefore, the efficiency and limit of detecting are not equivalent to those of standard kits for molecular diagnostics. Due to this difference in reaction efficiency, not all samples previously diagnosed as SARS-CoV-2 positives are eligible for screening with these assays; therefore, samples must be selected to exclude those with a low viral charge. The reference we used to select the samples for the analysis of the presence of mutations was the DeCoV-19 Kit Triplex, in which the molecular marker “N2” was selected as a reference. The samples, previously diagnosed as SARS-CoV-2 positive in which the “N2” gene was detected with a Cq of 27, or lower value, were chosen to be analyzed in the mutations assays. This Cq represents a value around 4 x 10^4^ copies per reaction.

### Screening for Detection of SARS-CoV-2 Mutations in COVID-19 Patients

This study was performed in a total of 1,060 clinical samples of COVID-19 patients from the state of Jalisco, Mexico, which have been previously diagnosed (from January 11 to 25, 2021) in the Laboratory for the Diagnosis of Emerging and Reemerging Diseases (LaDEER) of the University Center for Health Sciences (CUCS), of the University of Guadalajara (UdeG), Mexico. The patients were confirmed with the DeCoV-19 Kit Triplex diagnostic kit. All selected samples had a Cq value ≤ of 27 for the “N2” marker. The 69/70 deletion assay was performed in 1,040 samples of COVID-19 patients, while the K417N assay and E484K/N501Y assay were performed in 378 and 517 samples, respectively.

## Results

### Discrimination of Mutant and Non-Mutant Sequences

#### 69/70 Deletion Assay

**Figure 2:**
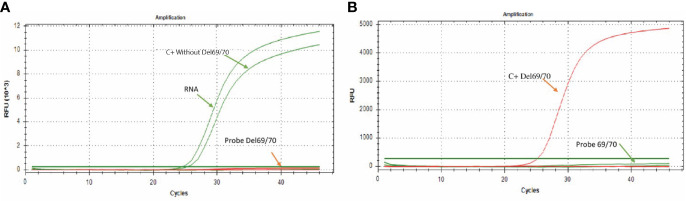
Amplification curves for the 69/70 assay. **(A)** The green color curve shows the amplification of the probe directed to the sequence without deletion (FAM) in a sample of SARS-CoV-2 positive patient; the curve of the probe directed to the sequence that presents the 69/70 deletion remains without signal of amplification. **(B)** The red curve shows the probe amplification directed to the 69/70 deletion (CFR 610) using the synthetic control containing the 69/70 deletion. In contrast, the probe directed to the sequence that does not present the deletion 69/70 remains without amplification signal.

Following the previously standardized conditions, it was observed that in the presence of RNA from a clinical sample or when adding the synthetic control C+ without-Del69/70 (control without deleted sequence), there was only amplification by the “Probe 69/70” probe, which is specific for sequences without deletion (**Figure 2A**); while in the presence of the synthetic control C + Del69/70 (control with deletion 69/70), the amplification was only observed by the “Probe Del 69/70” probe, which is specific for the detection of sequences with the 69/70 deletion (**Figure 2B**), allowing discriminate the presence or absence of this mutation in RNA samples.

#### K417N Assay

**Figure 3:**
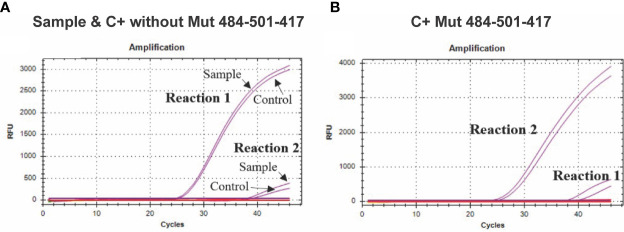
Amplification curves for the K417N assay. **(A)** The curves of four independent reactions are shown; the purple curves with a lower Cq value show the forward primer amplification directed to the sequence without K417N mutation (Reaction 1) in a sample of COVID-19 patients or control C+ without-Mut. In comparison, the signal with a higher Cq value shows the amplification when using the primer directed to the sequence that presents the mutation (Reaction 2), which presents an evident lag, in comparison to the first reaction. **(B)** The purple curves with a lower Cq value show the amplification of the forward primer directed to the K417N mutation (417N Fw) (Reaction 2) using synthetic control C+ Mut484-501-417 (which contains the mutation). In comparison, the amplification curve with a higher Cq value shows the amplification when using the primer directed to the sequence that does not present the mutation (Reaction 1) using the same control.

The assay aimed to detect the K417N mutation, discriminating between the sequences containing or not the mutation. While analyzing the amplification curves of the two reactions required for this assay, we observed amplification in both of them; however, there is a Cq lag between both curves caused by the specificity of the forward primer, which is specific for the sequences that present or do not present the mutation; thus, the reaction showing a curve with a lower Cq value indicates the type of sequence present in the reaction. For example, using the synthetic control C + without-Mut484-501-417 (without the K417N mutation) or RNA from clinical samples, the reaction that contains the primer that is 100% complementary to the sequences that do not present the K417N mutation showed a lower Cq value. In contrast, reaction 2, which contains the primer 100% complementary to the sequences that contain the mutation, presented a significantly higher Cq value, thus, indicating that the sequences present in reaction one did not present the target mutation (**Figure 3A**). On the other side, in the presence of the synthetic control presenting the mutation (C+ Mut484-501-417), reaction 2 showed a lower Cq value (**Figure 3B**).

#### E484K/N501Y Assay

**Figure 4:**
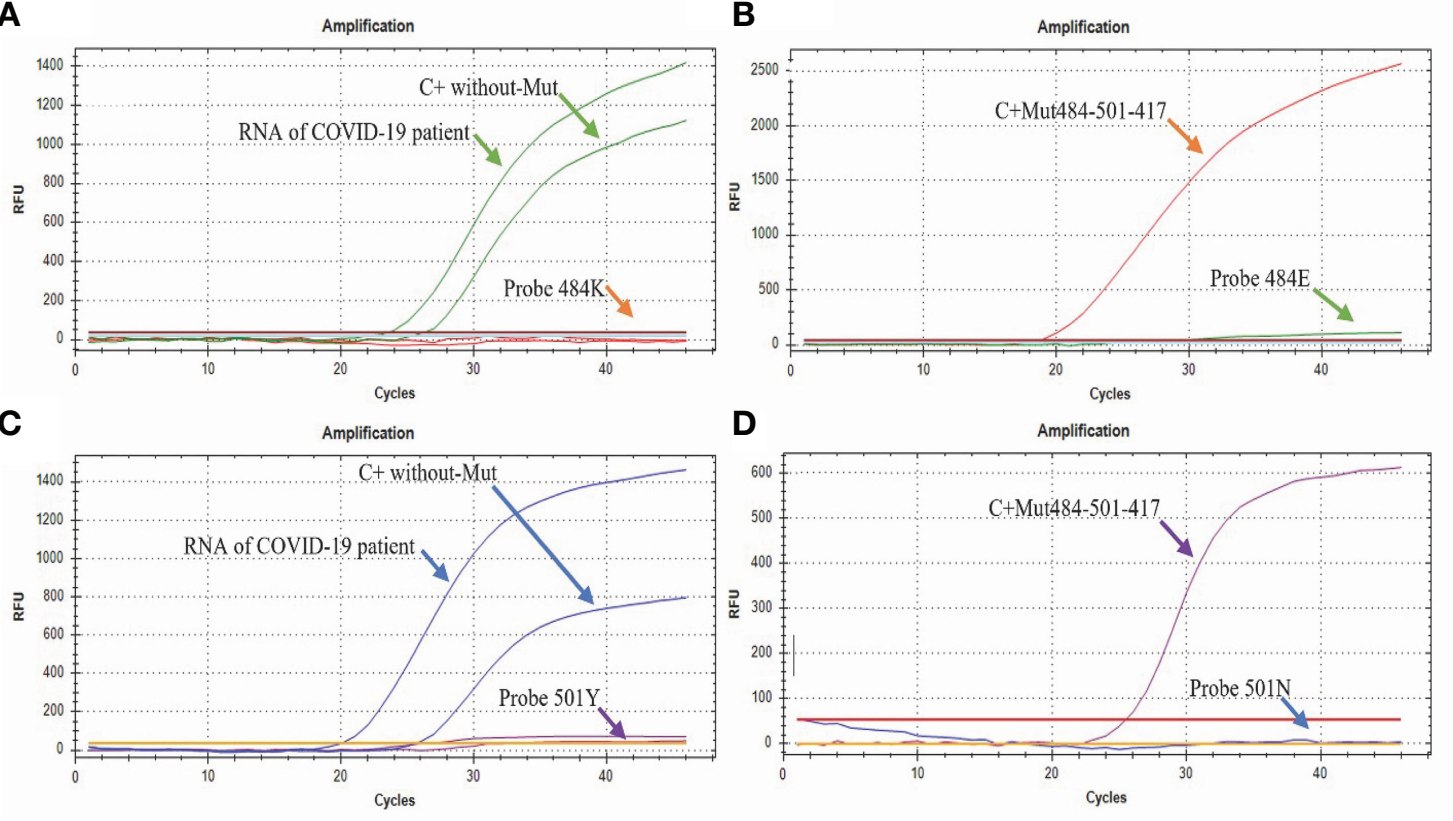
Amplification curves of the E484K/N501Y Assay. **(A)** The green curve shows the amplification of the probe directed to the sequence without the E484K mutation (FAM), while the signal by the probe directed to the sequence that presents the mutation remains without amplification signal; **(B)** the orange curve shows the amplification of the probe directed to the E484K mutation (CFR 610), while the probe directed to the sequence without mutation remains without amplification signal; **(C)** the blue curve shows the amplification of the probe directed to the sequence without mutation N501Y (HEX), while the probe directed to the sequence with the mutation remains without amplification signal; **(D)** the purple curve shows the amplification of the probe directed to the mutation N501Y (Quasar670) with the control containing the mutation (C+ Mut484-501-417), while the probe directed to the sequence without the mutation remains without amplification signal.

The assay easily discriminates between the sequences that contain or do not contain the mutations in a single quadruplex reaction. The assays performed in the presence of the sequence without the E484K mutation (**Figure 4A**) only showed amplification in the sequence designed for its detection (Probe 484E) either by adding the COVID-19 patient sample or the synthetic control (C + without-Mut484-501-417). On the other hand, in the presence of the synthetic control C+ Mut484-501-417 (with the E484K mutation) only the amplification by the 484K probe was observed, specific for detecting the mutation (**Figure 4B**).

Regarding the N501Y mutation, in the presence of the sequence without this mutation (**Figure 4C**), we only observed amplification by part of the sequence designed for its detection (Probe 501N HEX) either by adding the RNA from a clinical sample or the synthetic control C + without-Mut484-501-417. In contrast, in the presence of the synthetic control “C+ Mut484-501-417” (that presents the N501Y mutation), only amplification by the “Probe 501Y” probe was observed, which is specific for detection of the mutation (**Figure 4D**).

Representative amplicons from each assay were sequenced using Sanger sequencing to corroborate the absence or presence of the mutations of interest.

### Detection of SARS-CoV-2 Mutations in COVID-19 Patients From Jalisco, Mexico

**Figure 5:**
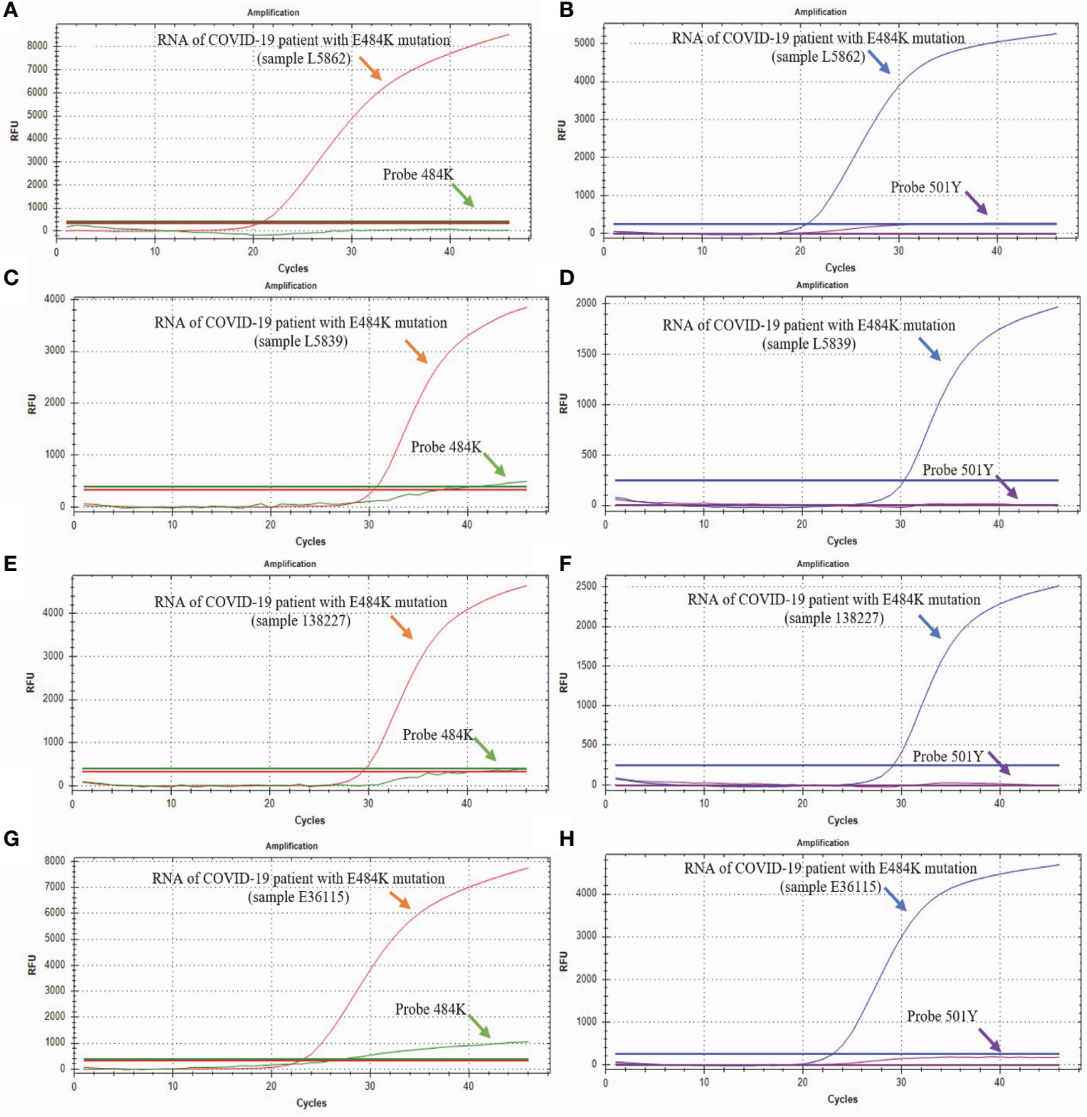
Detection of E484K and N501Y mutations. The green curve corresponds to the probe that detects the sequence without the E484K mutation (Probe 484E); the red curve corresponds to the probe that detects the sequence with the E484K mutation (Probe 484K); the blue curve corresponds to the probe that detects the sequence without the N501Y mutation, and the purple curve corresponds to the probe that detects the sequence with the N501Y mutation. **(A, B)** show the results from the L5862 patient; **(C, D)** show the results from the L5039 patient; **(E, F)** show the results from the 138227 patient; **(G, H)** show the results from the E36115 patient.

Through the analysis of 517 samples, we detected 9 positive samples for the E484K mutation, indicating a prevalence of 1.74% in Mexican patients from this study, diagnosed from January 11 to February 12, 2021. We could not detect the other three mutations (69-70del, K417N, and N501Y) in any patient. **Figure 5** shows four representative RT-qPCR curves from four cases with the E484K mutation. All 9 samples detected as E484K mutants had similar RT-qPCR curves.

**Figure 6:**
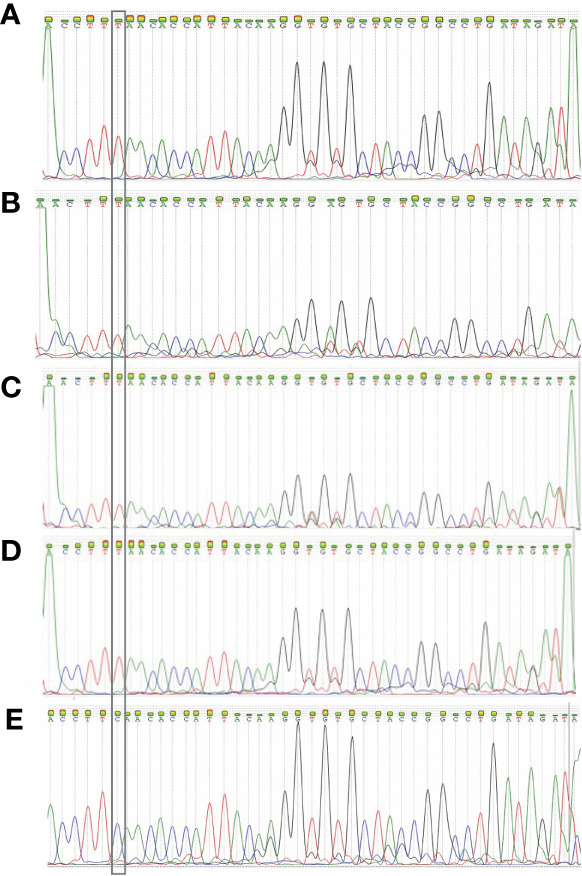
Electropherograms obtained from the Sanger sequencing of four samples. **(A)** 150441 patient; **(B)** 150 450 patient; **(C)** 138227 patient; **(D)** 139093 patient; **(E)** L782 patient. The first 4 samples show the E484K mutation, and the fifth is a wild-type sample. The inset highlights the position of the mutation, which shows that the original base “C” changed to a “T”.

After detecting the samples with the RT-qPCR assay, amplified sequences were sequenced with the Sanger method. The following 4 electropherograms show the sequencing results of four representative samples of samples with the E484K mutation. Also, an electropherogram of a sample without mutations was placed at the end to compare them (**Figure 6**). The rest of the electropherograms are shown in **Supplementary Material 1**. Lastly, the genomes of the viruses from those samples were sequenced and deposited in the NCBI database, with the following GenBank accession numbers: MW884227, MW884226, MW884225, MW884224, MW884223, MW884222, MW884221, MW884220, and MW884219.

The clinical characteristics of those patients with samples containing the E484K mutation are described in **Table 4**. The ages of patients ranged from 16 to 78 years; 4 men and 5 women. None of them reported signs of severe COVID-19, and six of them declared not having had a trip out of the city at least 30 days before the infection; only one patient reported a visit to a tourist port in the same state, which receives international tourists (Puerto Vallarta, Jalisco, Mexico) and two patients lived in this same city. None of the ten patients were in contact with each other.

**Table 4:** Clinical characteristics of COVID-19 patients with presence of SARS-CoV-2 E484K mutation.

Sample	Sex	Age	Travel history before the infection	Symptoms	Comorbidities or risk factor
150441	Female	78	Trip to a tourist area (Puerto Vallarta, Mexico)	Asymptomatic	Hypertension
150450	Male	67	Not reported	Asymptomatic	Not reported
138227	Male	60	Not reported	Headache, rhinorrhea, cough, general illness, muscle pain, and chest pain	Hypertension
139093	Male	37	Not reported	Headache and rhinorrhea	Smoking
157218	Male	35	Not reported	Fever, Headache, cough, shivers, myalgias and arthralgias, and dizziness	Obesity and Alcoholism
157231	Female	16	Not reported	Headache, cough, irritability, myalgias, arthralgias, dizziness, rhinorrhea, and anosmia	Not reported
E39931	Female	34	Not reported	Fever, Headache, cough, shivers, myalgias, and arthralgias, general discomfort, rhinorrhea, conjunctivitis, anosmia, ageusia	Immunosuppression
133706	Female	72	She works and lives in a tourist area (Puerto Vallarta, Mexico)	Headache, cough, irritability, myalgias, arthralgias, general discomfort, rhinorrhea	Hypertension, Fibromyalgia
145365	Female	19	She works and lives in a tourist area (Puerto Vallarta, Mexico)	Headache, cough, odynophagia, general discomfort, dizziness, conjunctivitis, anosmia, ageusia	No reported

## Discussion

In the present study, four molecular assays that detect SARS-CoV-2 mutations were developed. All assays were precise and robust, as shown by the assessment variability of the standard curves and Sanger sequencing. These assays will allow laboratories and countries to screen the mutations of clinical interest in samples for SARS-CoV-2 in a rapid and cost-effective format.

Since the RT-qPCR technique is more affordable than other molecular techniques such as sequencing, these assays could be implemented more easily in most countries, especially developing ones. We suggest that this approximation could be implemented as a screening strategy for detecting these SARS-CoV-2 mutations with pathogenic biological effects. It could offer several epidemiological advantages over the current countries’ strategies, relying upon samples being referred to specific institutions for their sequencing. This approach has been reported previously to detect other virus mutations with clinical importance, such as the ones in the influenza virus (Bolotin et al., 2009; van der Vries et al., 2010; Wong et al., 2011; Hoang Vu Mai et al., 2019). A similar screening was also performed by analyzing SARS-CoV-2 mutations and evaluating their diagnosis implication (Hernández-Huerta et al., 2020).

This study shows the potential of RT-qPCR as a quick and efficient strategy of molecular epidemiology in developing countries such as Mexico. Through this directed approach, we were able to find the E484K mutation in our country, and this is a starting point to select these specific cases and study them in greater depth through sequencing. It also allowed us to send these samples to the National Institute of Diagnostics and Epidemiology Reference (INDRE) “Dr. Manuel Martínez Báez” (INDRE, by its acronym in Spanish) of Mexico for their epidemiological monitoring using NGS to obtain the complete sequence genome. The full genome sequence showed the identification of the P.2 lineage through phylogenomic analysis in four samples whereas this E484K was detected with our RT-qPCR screening assay. This variant was first detected in Brazil on April 2020 and it had spread across this country. Actually, P.2 linage has been reported in 20 countries (PANGO lineages), and now Mexico will be added to this list. The spread and establishment of this lineage in several countries could be an alarm signal that warrants the intentional search for this variant in other nations.

This finding is important because in Mexico, previously, Hernández-Huerta et al., only identified the D614G mutation in the spike protein and the L84S mutation in the *ORF8* gene (Hernández-Huerta et al., 2020) and Taboada et al., only reported that the lineages circulating in Mexico changed from late February to March from A2 to B1 (Taboada et al., 2020) but both two studies did not found new lineages establishment in this country. Therefore, after our finding, a molecular epidemiological analysis is necessary in Mexico to determine the frequency and degree of spread of the P.2 variant.

The propagation of new lineages and the identification of viral mechanisms to overlap immune response are important topics for public health policies. The mutation E484K, first identified in march 2020 and then identified as a part of 501Y.V2 (B.1.351) and 484K.V1 (P.1) SARS-CoV-2 variants, has now been identified in the UK fast-spreading variant, prompting fears that the virus is evolving further and could become resistant to vaccines (Wise, 2021). In Brazil, this mutation has composed different lineages in a short time, and recently, it was identified in a sample from a reinfected patient (Nonaka et al., 2021). The E484K mutation is found within the RBD (a major target of neutralizing antibodies elicited during the primary exposure to SARS-CoV-2); thus, this mutation has been predicted to affect antibody neutralization. Therefore, that finding has caused several concerns related to this mutation and the risk of reinfection due to the possibility of escaping from neutralizing antibodies (Ferrareze et al., 2021).

Finding mutations with a clinical impact such as E484K might have significant implications for public health policies, surveillance, and immunization strategies; therefore, we consider that the world’s health authorities should consider the intentional search for mutations with important biological effects.

The limitation of our assays is the viral load required since clinical samples with viral load Cq value less than or equal to 27 are needed for optimal detection. Although this limitation represents a weakness for analyzing clinical samples, it is not a strong disadvantage considering the main objective of these assays, which is to analyze samples more conveniently, in terms of money and time to keep molecular epidemiological surveillance in near-real-time. Therefore, these assays could provide information that helps prevent and control future outbreaks generated by the introduction of variants in new geographical locations. Also, this method can be updated to include new emerging variants of clinical or epidemiological interest. The current assay conditions and limits can be further optimized, through the modification of current primers and probes, with chemical modifications such as LNA, MGB, ZNA, PNA, or HNA, could improve the efficiency of the assay, thus, allowing samples with a lower viral charge to be screened through this method.

In conclusion, this manuscript describes the development and validation of RT-qPCR assays to detect some mutations of interest at this pandemic period. The impact of these assays was demonstrated by finding a mutation of interest in the Mexican population, which is reported for the first time through this rapid molecular screening, which also allowed the identification of the P.2 lineage in Mexico.

## Data Availability Statement

The datasets presented in this study can be found in online repositories. The names of the repository/repositories and accession number(s) can be found in the article/**Supplementary Material**.

## Ethics Statement

The studies involving human participants were reviewed and approved by The research ethics committee of the University Center for Health Sciences, University of Guadalajara (approval number CI-00821). The patients/participants provided their written informed consent to participate in this study.

## Author Contributions

NV-M, R-SS, AV-L, OG-G, and JFM-V designed the study. RV-Z, B-GO, MD-S, MG-C, and GMO worked on the RT-qPCR experimental assay and sequencing. JH-B wrote the article. All authors contributed to the article and approved the submitted version.

## Funding

This research was supported by University Center for Health Sciences, University of Guadalajara, Mexico and ADN SINTÉTICO SAPI de CV.

## Conflict of Interest

Authors RS-S, AV-L, RV-Z, BG-O, MD-S, and OG-G have a working relationship with Genes2Life SAPI de CV.

The remaining authors declare that the research was conducted in the absence of any commercial or financial relationships that could be construed as a potential conflict of interest.
