# Supplementary figures and images for: RT-qPCR Assays for Rapid Detection of the N501Y, 69-70del, K417N, and E484K SARS-CoV-2 Mutations: A Screening Strategy to Identify Variants With Clinical Impact
Source: Front Cell Infect Microbiol. 2021 May 20;11:672562. doi: 10.3389/fcimb.2021.672562 (PMC8195289; doi:10.3389/fcimb.2021.672562)

Sample 157231

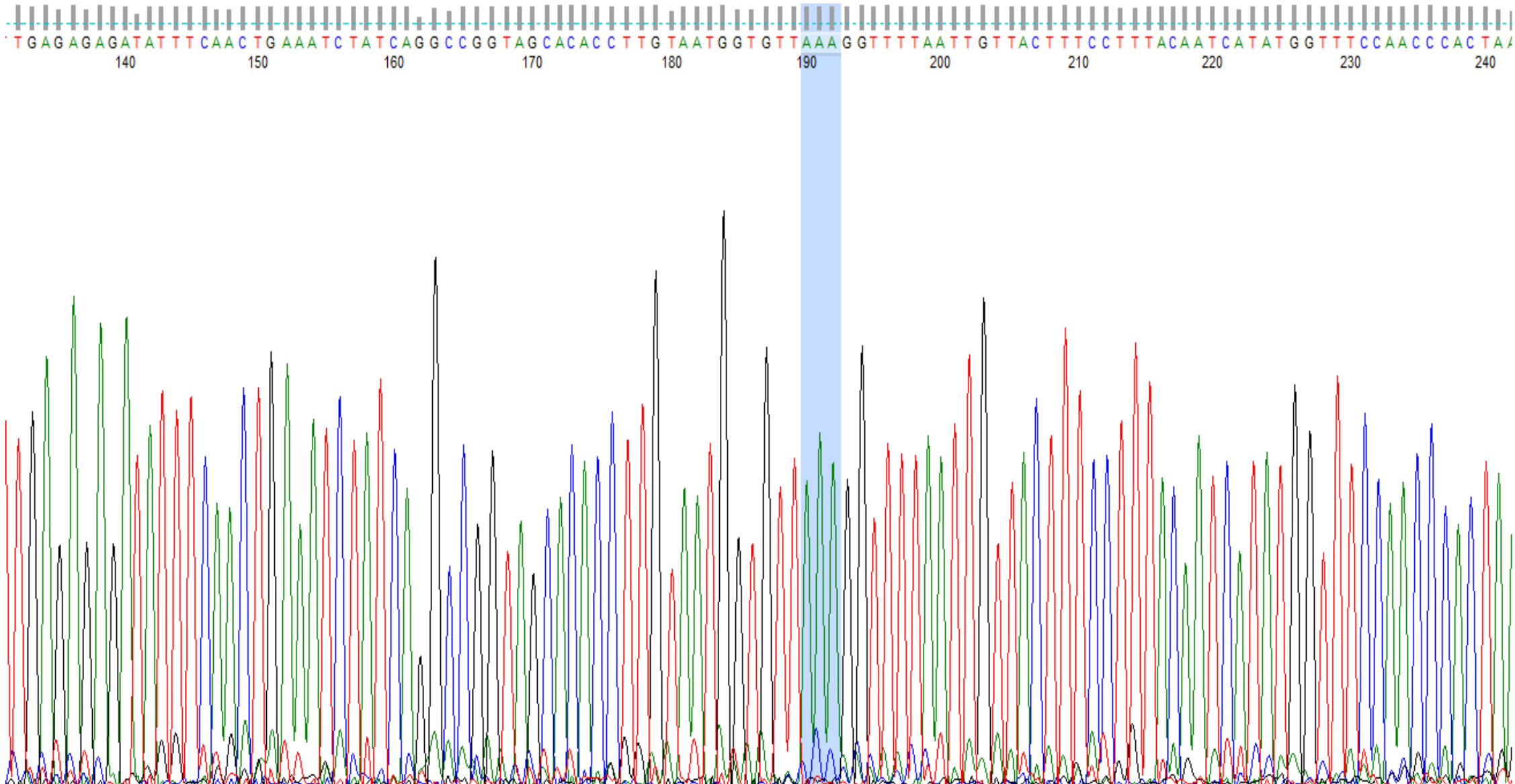

Sample E39931

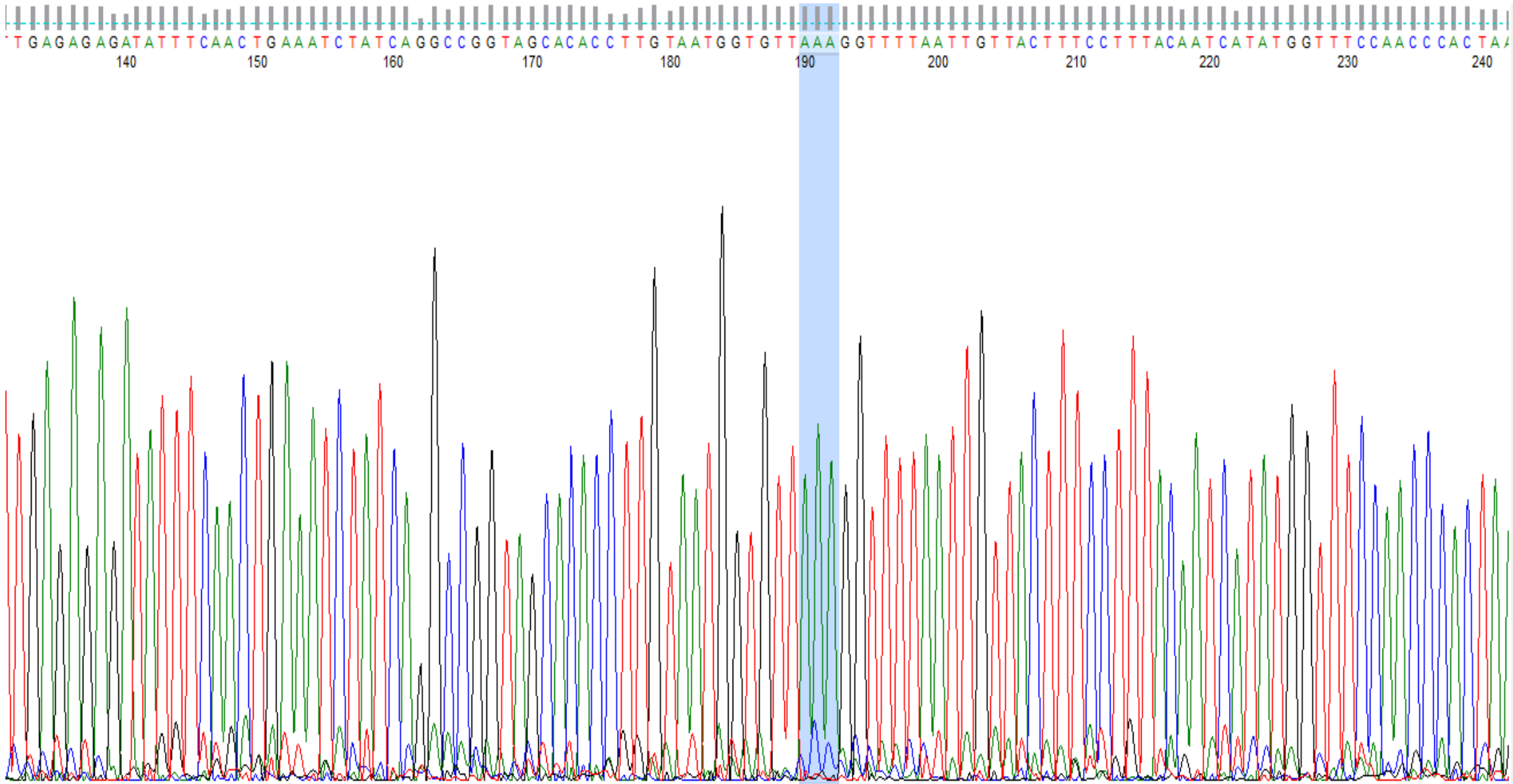

Sample 133706

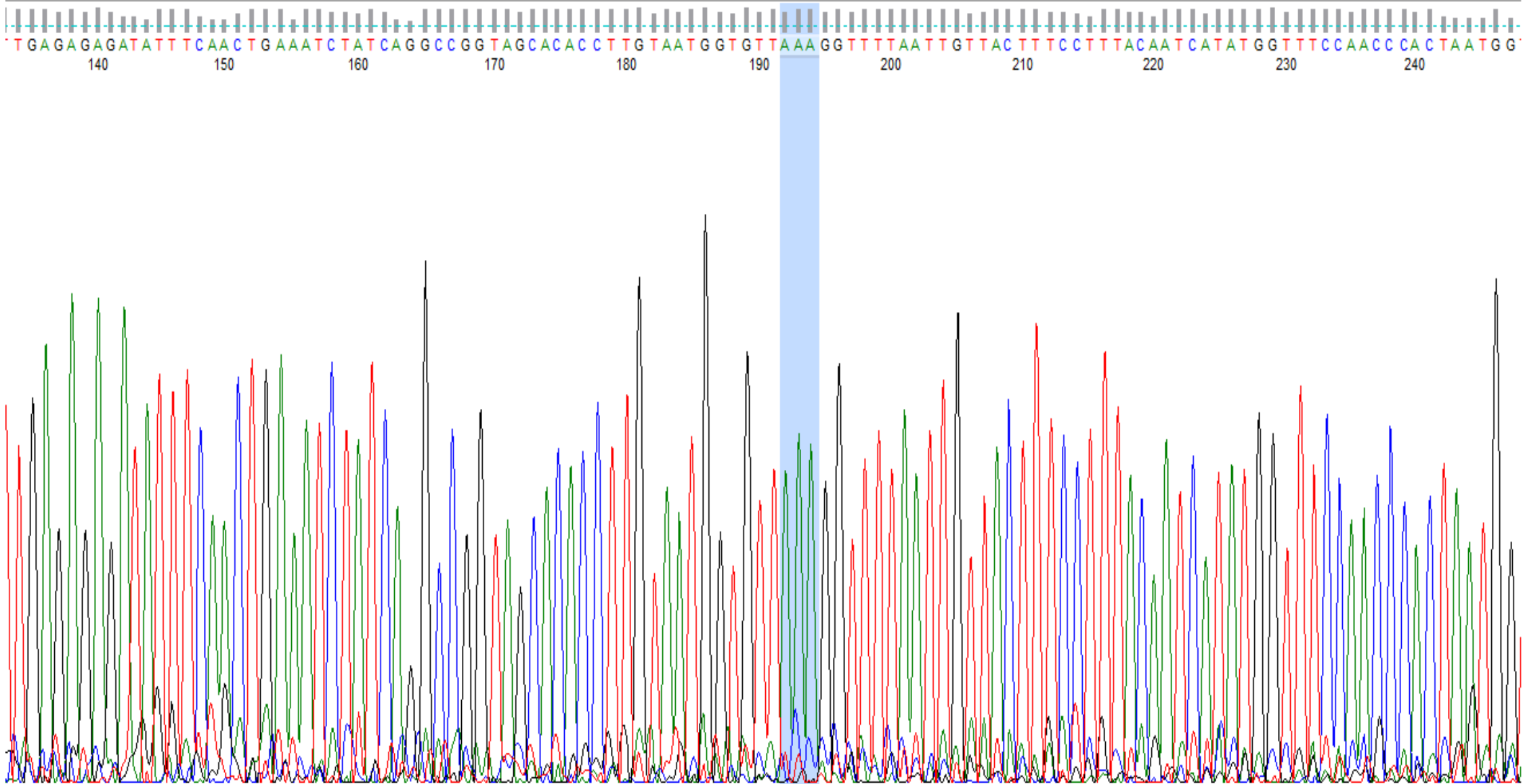

Sample 145365

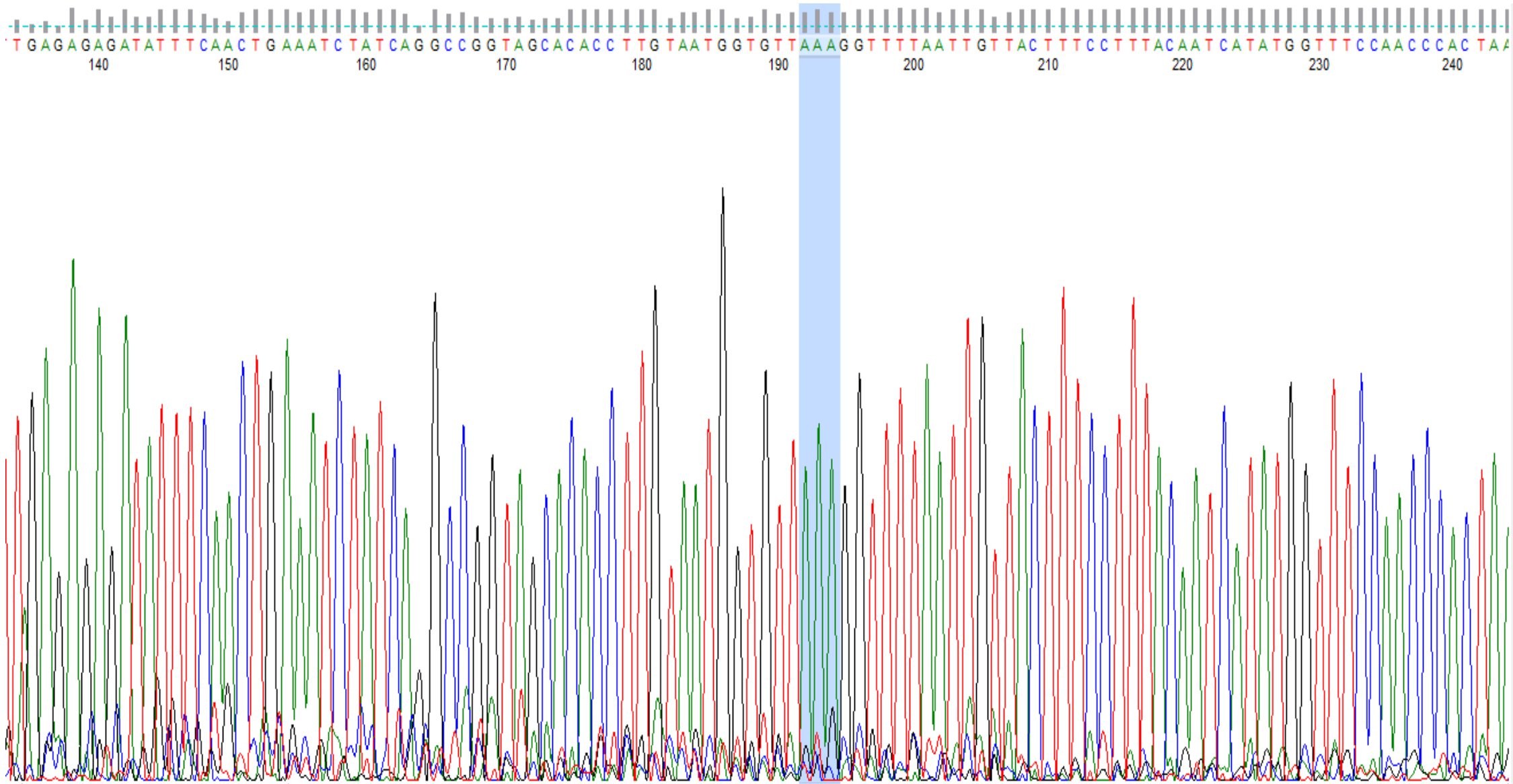

Sample 157218

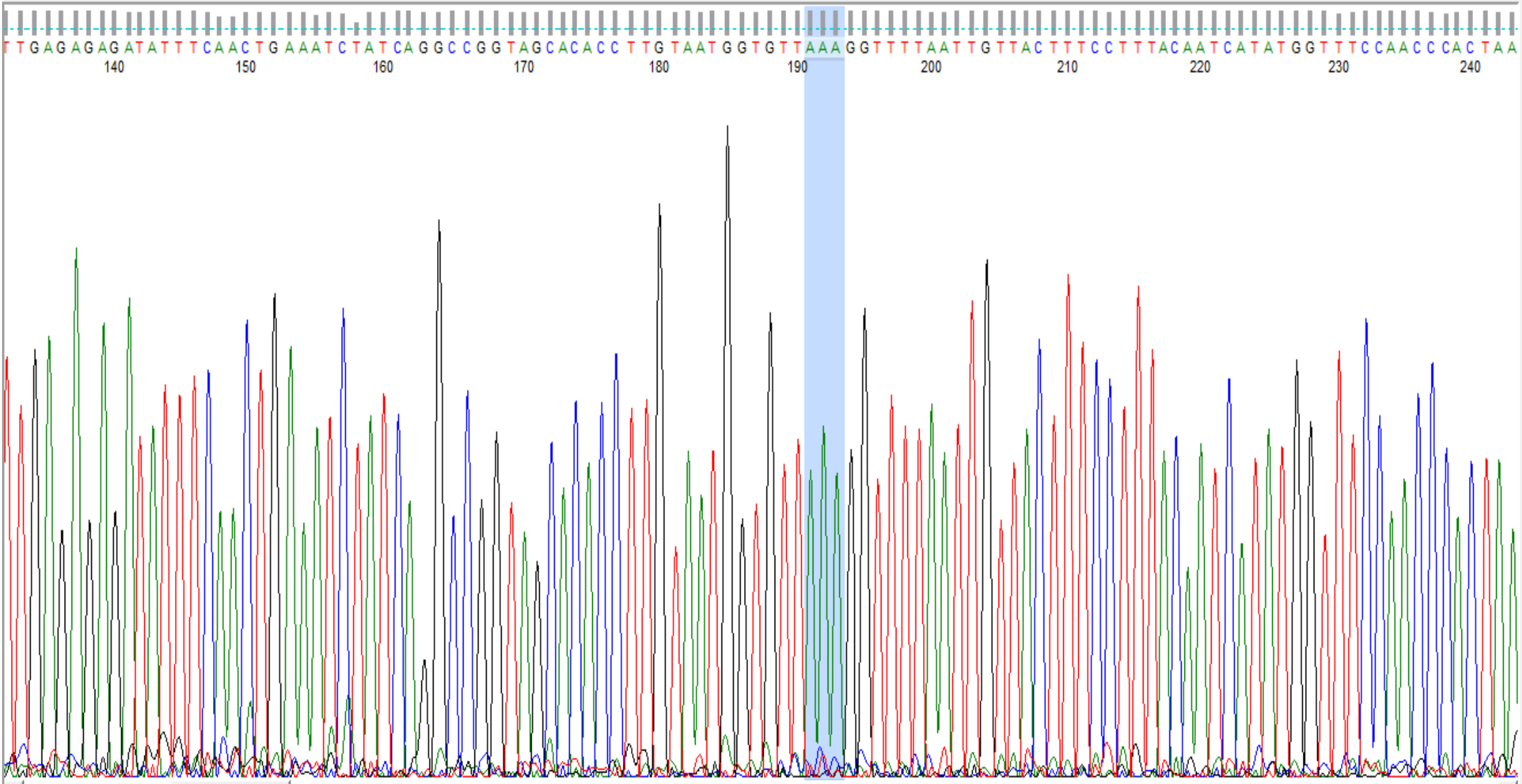

Supplement: Supplementary file 1 [file DataSheet_1.pdf]
